# Relative Telomere Length in Peripheral Blood Cells and Hypertension Risk among Mine Workers: A Case-Control Study in Chinese Coal Miners

**DOI:** 10.1155/2020/5681096

**Published:** 2020-12-03

**Authors:** Sheng-nan Yu, Shi-qi Chen, Guo-quan Fan, Wei-zhe Pan, Jin Jia, Qian Wang, Li Ma, Ben Li, Mei Qiang, Yu-lan Qiu, Tong Wang

**Affiliations:** ^1^Department of Toxicology, School of Public Health, Shanxi Medical University, Taiyuan, China; ^2^Department of Immunology, School of Basic Medical, Shanxi Medical University, Taiyuan, China; ^3^Department of Child and Adolescent Health, School of Public Health, Shanxi Medical University, Taiyuan, China; ^4^Department of Statistics, School of Public Health, Shanxi Medical University, Taiyuan, China

## Abstract

Hypertension is a common chronic disease in middle-aged and elderly people and is an important risk factor for many cardiovascular diseases. Its pathogenesis remains unclear. Epidemiological studies have found that the loss of telomere length in peripheral blood cells can increase the risk of coronary heart disease, myocardial infarction, and other diseases. However, a correlation between loss of telomere length and hypertension has not been established. In this study, we aimed to explore the association between telomere length and the risk of essential hypertension (EH) in Chinese coal miners. A case-control study was performed with 215 EH patients and 222 healthy controls in a large coal mining group located in North China. Face-to-face interviews were conducted by trained staff with the necessary medical knowledge. Relative telomere length (RTL) was measured by a quantitative real-time PCR assay using DNA extracted from peripheral blood. In the control group, the age-adjusted RTL was statistically significantly lower in miners performing hard physical labour compared with nonphysical labour (*P* = 0.043). A significantly shorter age-adjusted RTL was found in the control group of participants who consumed alcohol regularly compared with those who do not consume alcohol (*P* = 0.024). Age-adjusted RTL was negatively correlated with body mass index (BMI) and alcohol consumption. Hypertension was also found to be significantly correlated with factors such as age, BMI, alcohol consumption, smoking, and tea consumption. Our results suggest that RTL is associated with hypertension in coal miners.

## 1. Introduction

Hypertension is a chronic disease commonly observed in middle-aged and elderly people and is an important risk factor for many cardiovascular diseases [1]. More than 25% of the Chinese population suffer from hypertension [2, 3], resulting in serious health implications within the population, including occupational populations in mining areas [4]. In 2015, health examinations performed in accordance with the Enhanced Coal Workers' Health Surveillance Program (ECWHSP) found that obesity and hypertension were more common in coal-exposed individuals than in unexposed individuals [5]. Recent epidemiological investigations showed that the incidence of cardiovascular disease, especially hypertension, in coal miners is higher than that in the general population [6]. Another study also showed that approximately 50% of mining-excavator operators developed temporary hypertension within a decade [7].

The telomere is a highly regulated and dynamic complex found at the end of chromosomes consisting of a tract of tandem repeated short DNA repeats and associated protective proteins [8]. Since telomeres progressively shorten with increased turnover and chronological age in dividing somatic cells, telomere length may also change and have functional roles in normal and pathophysiological processes [9]. Telomere length is regulated by genetic and environmental factors, and changes in adult telomere length occur chronically over time. Therefore, telomere length is regarded as a marker for biological ageing and age-related diseases such as tumors [10–12]. EH is also an ageing-related chronic pathological condition. Animal studies and epidemiological surveys found that short telomeres may play an important role in the aetiology of hypertension [13–15]. A recent study found that occupational exposure to coal is positively associated with shorter telomere length [16], but the relationship between telomere length and high blood pressure among coal miners is unclear. Shanxi Province is rich in coal resources and has a large number of coal miners. Their health status cannot be ignored.

The aim of this study was to illustrate the role of RTL in occupational coal workers suffering from EH. Additionally, this study also addresses other influencing factors related to EH in order to promote the health of the workers.

## 2. Materials and Methods

### 2.1. Study Population and Sample Collection

A large coal mine in the northern Shanxi Province was selected as the research site. A total of 437 coal miners (the average age was 43.97 ± 8.90) were recruited, of which 215 (44.00 ± 8.61) have EH and 222 (43.95 ± 9.18) were healthy. Questionnaires [17] were administered in the form of face-to-face interviews. The survey included gender, date of birth, education, marital status, place of work, working hours, work intensity, smoking status (current smokers and nonsmokers; current smoking was defined as smoking more than 1 cigarette per day, continuous or cumulative for more than 6 months), tea consumption, and alcohol consumption (consumption was defined as drinking more than once a week and drinking continuously for more than six months). The inclusion criteria of the study people were as follows. (1) The people were aged 18-65 years and had worked in the large coal mine for ≥1 year; case group: diagnosed as essential hypertension; control group: no hypertension. (2) The people participated voluntarily and signed an informed consent. Exclusion criteria were as follows: (1) patients with Parkinson's disease, tumor, and kidney disease (diseases affecting telomere length) at the time of investigation; (2) people with secondary hypertension; and (3) pregnant women or those with incomplete information. Blood pressure was measured after 10 minutes of rest. EH was diagnosed according to the “China Guidelines for the Prevention and Treatment of Hypertension 2010” as systolic blood pressure (SBP) ≥ 140 mmHg and diastolic blood pressure (DBP) ≥ 90 mmHg. In accordance with the Chinese guidelines on the prevention and control of overweight and obesity in adults, body mass index (BMI) was used to classify thin (BMI < 18.5), normal (18.5 ≤ BMI < 24), overweight (24 ≤ BMI < 28), and obese (BMI ≥ 28.00) participants. Blood was taken 12 hours after fasting to obtain a DNA separation sample.

### 2.2. Laboratory Analysis

Peripheral blood samples were collected in ethylenediaminetetraacetic acid tubes after overnight fasting (>12 h) and kept at −20°C before use. Genomic DNA was isolated from 200 *μ*l of blood using the QIAamp DNA Blood Mini Kit (QIAGEN, Valencia, CA, USA) following the manufacturer's instructions [18]. In brief, the samples were thawed on ice and mixed briefly by vortexing. Then, the plasma samples were incubated with lysis buffer and proteinase K at 56°C for 10 min. At the final step of isolation, DNA was eluted with 150 *μ*l of nuclease-free deionized and distilled H_2_O. The quantity and purity of the DNA were assayed using a NanoDrop 2000 spectrophotometer (Thermo Scientific, Wilmington, DE, USA), and all DNA samples had OD260/OD280 values of 1.7–2.1. The DNA samples were stored at −80°C until further use. RTL was quantified by real-time polymerase chain reaction (PCR) as described in previous studies [19, 20]. The primer sequences Tel-F, 5′-CAGCAAGTGGGAAGGTGTAATCC-3′, and Tel-R, 5′-CCCATTCTATCATCAACGGGTACAA-3′, were used to measure the RTL, while the primers 36B4-F, 5′-GCTTCTGACACAACTGTGTTCACTAGC-3′, and 36B4-R, 5′-CACCAACTTCATCCACGTTCACC-3′, were used to amplify the single-copy nuclear 36B4 gene. The assay was performed using the Maxima SYBR Green qPCR Master Mix (TAKARA, Dalian, China) supplied by Line Gene 9660 Real-Time Detection Systems (Bioer, Hangzhou, China). Quantitative PCR was performed under the following conditions: denaturation at 95°C for 10 min, followed by 40 cycles of 10 s at 95°C, 30 s at 60°C, and 30 s at 72°C. All assays were carried out in triplicates using 20 ng of DNA per 10 *μ*l of reaction mixture. The acceptable standard deviation (s.d.) of the triplicate threshold cycle (Ct) values was set at 0.3. If the result was out of the acceptable range, then the run was repeated for the same sample. RTL was calculated using the following equation: −ΔCt (ΔCt = Ct_Tel_ − Ct_36B4_).

### 2.3. Statistical Analysis

Data were double-entered into Epi Info version 3.5.1 (CDC, Atlanta, GA, USA), which reduces error in creating electronic data sets before statistical analysis. The difference in the distribution of characteristics between the cases and the controls was evaluated using the chi-square test for categorical variables (age cohorts, education status, work type, work duration, workplace, BMI, alcohol consumption, smoking status, and tea consumption). One-way ANOVA and multiple regression analysis were used to reveal factors that influence RTL in the controls. Crude odds ratios (ORs) and 95% confidence intervals (CIs) for each of the known risk factors were obtained through univariate logistic regression analysis to evaluate their association with the risk of hypertension. The association between the risk of EH and RTL was estimated using OR and 95% CIs in unconditional multivariate logistic regression analysis after adjustment for age, education status, work type, work duration, workplace, BMI, alcohol consumption, smoking status, and tea consumption, where appropriate. In the regression models, RTL was analysed as a categorical variable based on a cutoff point at the median values of the controls or continuous variables. All statistical analyses were performed with the Statistical Package for the Social Sciences (SPSS, version 24.0 for Windows). All *P* values reported were two-sided and considered statistically significant at *P* < 0.05.

## 3. Results

### 3.1. Characteristics of the Participants with and without EH

There were no significant differences in age, gender, education, work type, workplace, and tea consumption between the EH group and the control group (*P* > 0.05). However, there were significant differences in work duration, BMI, alcohol consumption, current smoking, and RTL (*P* < 0.05), as shown in Table 1.

### 3.2. The Relationship between RTL and Age

**Figure 1:**
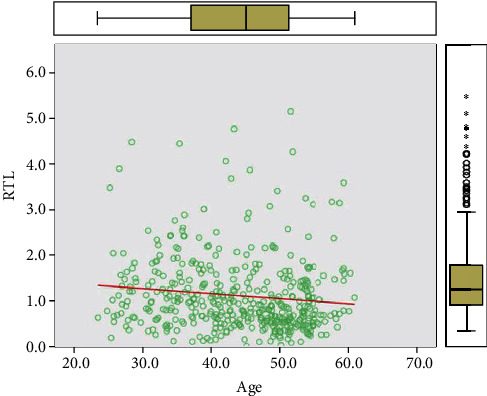
Relationship between telomere length and age in the total population. RTL: relative telomere length.

A total of 437 peripheral blood samples from EH and healthy subjects were collected from participants aged between 18 and 65 years. The RTL of peripheral blood cells was determined by RT-PCR. The average RTL was 1.1236 ± 0.8100 and was significantly negatively correlated with age (*P* < 0.05), indicating that telomeres shortened with age as shown in Figure 1.

**Figure 2:**
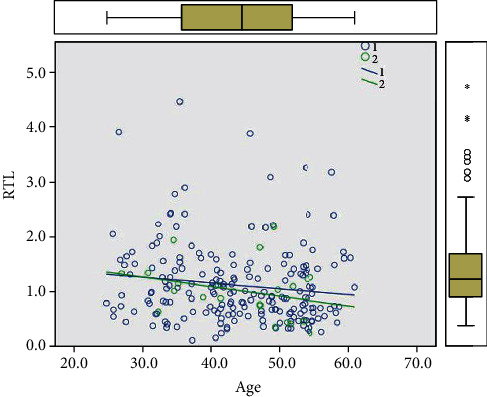
Relationship between telomere length and age in the control group: 1: male; 2: female.

The healthy group included 222 participants (200 males and 22 females) aged from 24 to 61 years old (43.95 ± 9.18) with an average peripheral blood cell RTL of 1.106 ± 0.689. The Pearson correlation analysis showed a significant negative correlation between RTL and age in the control group. RTL was negatively correlated with age in both males and females. The male telomere length shortening rate was -0.01 with an *R*^2^ value of 0.019, while the female telomere length shortening rate was -0.017 with an *R*^2^ value of 0.088. The rate of telomere shortening in the control group was higher in females than in males as shown in Figure 2. The EH group included 215 participants (194 males and 21 females) aged from 23 to 61 years old (44.00 ± 8.61) with an average peripheral blood cell RTL of 1.142 ± 0.920. The Pearson correlation analysis showed no significant correlation between RTL and age in the EH group.

### 3.3. Influencing Factors of Age-Adjusted RTL in the Control Group

In order to compare the effects of different factors on RTL, using age as a variable, regression analysis was performed on the dependent variable and obtains the residual after standardization as the age-adjusted RTL. After adjusting for age, one-way ANOVA showed that work type and alcohol consumption had significant effects on age-adjusted RTL (*P* < 0.05), while gender, education, work duration, workplace, BMI, current smoking, and tea consumption had no significant effect as shown in Table 2.

### 3.4. Multiple Regression Analysis of Age-Adjusted RTL

Multiple linear regression analysis of the combined effects of gender, BMI, dietary habits, and other factors on age-adjusted RTL was performed. The results indicated that body mass index (*P* < 0.01), alcohol consumption (*P* < 0.05), and age-adjusted RTL were negatively correlated, while current smoking (*P* < 0.05) was positively correlated with age-adjusted RTL (Table 3). Gender, education, tea consumption, and other vocational factors, including work type, work duration, and workplace, were not significantly associated with age-adjusted RTL.

### 3.5. Univariate Logistic Regression of EH and Other Variables

As outlined in Table 4, univariate logistic regression analysis of 11 factors including age and gender showed that the risk of EH gradually increases with increasing BMI. The risk of EH in people whose BMI was between 24 to 28 and more than 28 was 11.484 times and 27.077 times more than those whose BMI was less than 18.5(*P* < 0.05), respectively. The risk of EH was also significantly different in alcohol consumption; the risk of EH was 2.313 times higher in people who drink wine than those who do not (*P* < 0.05). Current smokers have 1.791 times higher risk of hypertension than nonsmokers. The population RTL varied from 0.0199 to 5.1419 and was divided into 3 levels according to the population distribution. The results show a significant difference between longer and shorter RTL. The risk of EH in people with a RTL of 0.57927 to 1.45126 was 0.478 times less than people whose RTL was less than 0.57927 (*P* < 0.05). The results indicate that telomere length is significantly associated with the risk of EH and that telomere length is a protective factor against EH.

### 3.6. Multifactor Logistic Regression of EH and Other Variables

A multifactor logistic regression analysis was performed to consider the interaction between various confounding factors, as shown in Table 5. The results showed that age, BMI, alcohol consumption, smoking (currently smoking), tea consumption, and age-adjusted RTL significantly affected the risk of EH. As BMI is a risk factor for EH, the risk of EH increased with increasing BMI. Alcohol consumption and smoking are also risk factors for EH; hence, the risk of EH in people who drink and smoke was 2.103 times and 1.812 times higher, respectively, than those who do not. The results also indicated that tea consumption is a protective factor against EH. The risk of EH in people who drink tea was 0.609 lower than in those who do not. An analysis of RTL as a categorical variable indicated that RTL may be a predictive factor against EH as the risk of EH increased with decreasing telomere length.

## 4. Discussion

In this case-control study, we investigated the influence of various factors on EH in coal mine workers, particularly the relationship between changes in the RTL in coal miners and EH. Previous studies have shown that the incidence of hypertension in workers is significantly higher than that in the general population [21, 22]. Souza et al. demonstrated that occupational coal exposure was associated with shortened RTL [16], and a case-control study in India found that shorter RTL was associated with essential hypertension [23]. The telomere length of health care workers (mainly nurses) showed that shift work can lead to shortened telomere length, increased systemic inflammation, and oxidative stress imbalance [24]. The shift work of coal miners in this study may also be an important risk factor for telomere length, which deserves further research. Our results suggest that RTL is associated with hypertension in coal miners. In our study, we divided RTL into three levels according to percentile pitch. Multivariate logistic regression analysis showed that the risk of hypertension in moderate RTL (0.57927–1.45126) is 0.529 times that in shorter RTL. This finding indicates that long RTL is a protective factor in hypertension, which is consistent with previous findings [1]. The reason coal miners have a higher risk of hypertension than the general population may be because the chemical composition of coal is complex and it is accompanied by occupational hazards such as productive dust and toxic heavy metals, toxic and harmful gases, and noise throughout the entire coal production process. Increasing evidence also indicated that toxic and harmful particles enter the body and produce an inflammatory response, which releases reactive oxygen species, leading to an imbalance between oxidation and antioxidant effects in the body, followed by oxidative stress [25]. Exposure to toxic heavy metals, which usually occurs in workers, increases the risk of cardiovascular disease by increasing homocysteine levels, the underlying mechanism may be that high homocysteine leads to changes in the elasticity of the vascular wall, the inhibition of nitric oxide synthesis, and the increase of oxidative stress [26]. A study in mouse models provided evidence that chronic inflammation causes telomere dysfunction and premature ageing [27]. Liu et al.'s study showed that noise is an independent risk factor for hypertension in coal miners [28]. An epidemiological investigation showed that noise can accelerate telomere length loss in peripheral blood leukocytes [29]. Noise, as an environmental stressor, can increase the level of glutathione and harm the body's antioxidant system balance [30]. DNA damage can also be a product of oxidative stress. In our study, the mean age and RTL of the non-EH group were 43.95 ± 9.18 and 1.106 ± 0.689, respectively. A Chinese study about omethoate showed that the mean RTL of unexposed control group ≤ 40 years old was 3.03 ± 0.35, and the mean RTL of >40 years old was 2.97 ± 0.37 [31]. The RTL of coal miners is shorter than that of the general population, probably due to the special exposure of the miner occupation. Based on the above information, a hypothesis can be put forward that toxic and harmful particles and noise will affect RTL and eventually lead to higher risk of hypertension in coal miners than the general population. Further research is needed to provide a molecular mechanism that affects telomere length and hypertension in coal miners. In this study, our result shows that the rate of shortening of RTL in healthy control women is significantly higher than that of men, and this conclusion is consistent with many studies [32, 33]. But there are also opposite conclusions [34], which may be caused by the age span of the study population. We also found that RTL was negatively correlated with age, meaning that RTL gradually shortened with age. Therefore, we should exclude age as a factor when studying RTL within a population. Age-adjusted one-way ANOVA of factors influencing RTL in the control group showed that work type and alcohol consumption had significant effects on age-adjusted RTL. Different work types result in different degrees of possible exposure to coal dust, which explains the influence of work type on age-adjusted RTL. The results of this study also demonstrate that BMI and alcohol consumption were negatively correlated with age-adjusted RTL, suggesting that a healthy BMI and limited alcohol consumption could slow down the rate of RTL shortening in coal miners. The results of multivariate logistic regression analysis showed that age, BMI, alcohol consumption, and current smoking were risk factors for hypertension. The association between BMI and hypertension is consistent with Williams' finding [35]. One study found that the rate of leukocyte telomere shortening is three times greater in active smokers than in nonsmokers. The leukocyte telomere length of previous smokers who had quit smoking before the study was also shorter than in those who never smoked [36]. Our research indicates that current smoking is a risk factor for hypertension, and smoking may cause the shortening of RTL and further induce hypertension. In other words, the shortening of RTL may be an internal influencing mechanism of hypertension. Further studies are required to validate this hypothesis. A systematic review and meta-analysis also found that alcohol consumption was closely related to hypertension, and reducing alcohol intake can effectively reduce blood pressure [37]. Tea consumption and RTL were protective factors which suggest that an active lifestyle intervention could reduce the incidence of hypertension [4]. The effect of tea consumption on blood pressure has been controversial [38], but our findings support the linear mixed effects model analysis which indicates that tea consumption has a positive effect on diastolic blood pressure [39]. We expected that the place of work within this occupational population may have an impact on hypertension and telomere length, but the results do not lead to this conclusion (*P* > 0.05). After further investigation, we found that the occupational groups working in the coal mine area are regularly rotated, which may explain this result.

It should be noted that this study has some limitations. First, this case-control study faces the same inherent limitations seen in retrospective studies such as recall bias and inferior timing of causal association. Second, real-time quantitative PCR was used to determine RTL due to its high specificity. Although high-throughput processing of large samples has been widely used, it has become controversial in recent years compared with the traditional DNA blotting because the result of the measurement is a relative value and not an absolute value. Additionally, the resolution and accuracy of this method is lower than those of the conventional method [40] and repeated measurements of the coefficient of variation exceeded 10%.

In conclusion, our results suggest that RTL is associated with EH in coal miners. We also found that the factors such as BMI, smoking, alcohol consumption, and tea consumption were closely associated with EH in coal miners. This information may provide a scientific basis for preventing hypertension through lifestyle interventions. It also provides a solid foundation to study the pathogenesis of hypertension in mine workers.

## Figures and Tables

**Table 1:** Characteristics of the participants with and without hypertension.

Characteristics	Case (*N* = 215)	Control (*N* = 222)	*χ* ^2^/*t*	*P* value
Age			0.969	0.809
≤30	18 (8.4)	16 (7.2)		
30~40	56 (26.0)	57 (25.7)		
40~50	79 (36.7)	76 (34.2)		
>50	62 (28.8)	73 (32.9)		
Gender			0.002	0.960
Male	194 (90.2)	200 (90.1)		
Female	21 (9.8)	22 (9.9)		
Education			3.787	0.285
College or higher	16 (7.4)	28 (12.6)		
Senior middle school	135 (62.8)	131 (59.0)		
Junior middle school	54 (25.1)	56 (25.2)		
Primary school and less than primary school	10 (4.7)	7 (3.2)		
Work type			3.397	0.183
Hard physical labour	52 (24.2)	71 (32.0)		
Light physical labour	113 (52.6)	102 (45.9)		
Nonphysical labour	50 (23.3)	49 (22.1)		
Work duration			16.018	0.007^∗^
≤1 year	5 (2.3)	6 (2.7)		
2~ years	18 (8.4)	21 (9.5)		
4~ years	36 (16.7)	70 (31.5)		
11~ years	27 (12.6)	26 (11.7)		
16~ years	28 (13.0)	27 (12.2)		
≥21 years	101 (47.0)	72 (32.4)		
Workplace			3.844	0.279
Underground	52 (24.2)	71 (32.0)		
Underground auxiliary	68 (31.6)	69 (31.1)		
Ground	55 (25.6)	46 (20.7)		
Office workers	40 (18.6)	36 (16.2)		
BMI			34.569	<0.001^∗^
<18.5	1 (0.5)	11 (5.0)		
18.5~24	55 (25.6)	94 (42.3)		
24~28	95 (44.2)	91 (41.0)		
≥28	64 (29.8)	26 (11.7)		
Alcohol consumption			17.628	<0.001^∗^
No	111 (51.6)	158 (71.2)		
Yes	104 (48.4)	64 (28.8)		
Current smoking			9.141	0.002^∗^
No	89 (41.4)	124 (55.9)		
Yes	126 (58.6)	98 (44.1)		
Tea consumption			0.192	0.661
No	138 (64.2)	138 (62.2)		
Yes	77 (35.8)	84 (37.8)		
RTL			10.685	0.005^∗^
<0.57927	66 (30.7)	44 (19.8)		
0.57927~1.45126	91 (42.3)	127 (57.2)		
>1.45126	58 (27.0)	51 (23.0)		

^∗^ *P* < 0.05 and the difference is statistically significant. Abbreviations: RTL: relative telomere length; BMI: body mass index.

**Table 2:** One-way ANOVA of influencing factors of age-adjusted RTL (control group).

Characteristics		*N* (%)	Age-adjusted RTL	*P* value
Gender	Male	200 (90.1)	−0.010 ± 0.869	0.531
	Female	22 (9.9)	−0.130 ± 0.611	
Education	College or higher	28 (12.6)	0.060 ± 0.903	0.937
	Senior middle school	131 (59.0)	−0.019 ± 0.892	
	Junior middle school	56 (25.2)	−0.064 ± 0.751	
	Primary school and less than primary schoolwork	7 (3.2)	−0.068 ± 0.492	
Work type	Hard physical labour	71 (32.0)	0.037 ± 0.822	0.043^∗^
	Light physical labour	102 (45.9)	−0.165 ± 0.724	
	Nonphysical labour	49 (22.1)	0.188 ± 1.057	
Work duration	≤1 year	6 (2.7)	0.268 ± 1.671	0.528
	2~ years	21 (9.5)	−0.273 ± 0.738	
	4~ years	70 (31.5)	−0.088 ± 0.646	
	11~ years	26 (11.7)	0.135 ± 1.222	
	16~ years	27 (12.2)	−0.021 ± 0.740	
	≥21 years	72 (32.4)	0.034 ± 0.840	
Workplace	Underground	71 (32.0)	−0.180 ± 0.614	0.080
	Underground auxiliary	69 (31.1)	−0.047 ± 0.849	
	Ground	46 (20.7)	0.040 ± 0.769	
	Office workers	36 (16.2)	0.258 ± 1.212	
BMI	<18.5	11 (5.0)	0.124 ± 0.689	0.338
	18.5~24	94 (42.3)	0.025 ± 0.884	
	24~28	91 (41.0)	−0.010 ± 0.893	
	≥28	26 (11.7)	−0.297 ± 0.518	
Alcohol consumption	No	158 (71.2)	0.059 ± 0.904	0.024^∗^
	Yes	64 (28.8)	−0.222 ± 0.647	
Current smoking	No	124 (55.9)	0.0145 ± 0.848	0.469
	Yes	98 (44.1)	−0.069 ± 0.847	
Tea consumption	No	138 (62.2)	−0.058 ± 0.748	0.414
	Yes	84 (37.8)	0.037 ± 0.989	

Values are expressed as mean ± standard deviation. ^∗^*P* < 0.05 and the difference is statistically significant. Abbreviations: RTL: relative telomere length; BMI: body mass index.

**Table 3:** Multiple regression analysis of age-adjusted RTL.

Characteristics	95% CI		*t*	*P* value	Standardized coefficient *β*
	Upper limit	Lower limit			
Gender	-0.802	0.025	-1.854	0.191	-0.138
Education	-0.251	0.129	-0.633	0.065	-0.050
Work type	-0.322	0.098	-1.051	0.527	-0.097
Work duration	-0.023	0.132	1.376	0.294	0.098
Workplace	0.065	0.351	2.868	0.170	0.262
BMI	-0.302	-0.009	-2.092	0.005^∗^	-0.140
Alcohol consumption	-0.578	-0.056	-2.392	0.038^∗^	-0.170
Current smoking	-0.193	0.299	0.423	0.018^∗^	0.031
Tea consumption	-0.134	0.347	0.870	0.673	0.061

^∗^ *P* < 0.05 and the difference is statistically significant. Abbreviations: RTL: relative telomere length; BMI: body mass index.

**Table 4:** Univariate logistic regression of EH and other variables.

Characteristics		Case	Control	*P* value	OR	95% CI	
		*n* (%)	*n* (%)			Upper limit	Lower limit
Age	≤30	18 (8.4)	16 (7.2)				
	30~40	56 (26.0)	57 (25.7)	0.729	0.873	0.405	1.882
	40~50	79 (36.7)	76 (34.2)	0.835	0.924	0.439	1.943
	>50	62 (28.8)	73 (32.9)	0.465	0.755	0.355	1.604
Gender	Male	194 (90.2)	200 (90.1)				
	Female	21 (9.8)	22 (9.9)	0.960	0.984	0.524	1.847
Education	College or higher	16 (7.4)	28 (12.6)				
	Senior middle school	135 (62.8)	131 (59.0)	0.080	1.803	0.932	3.488
	Junior middle school	54 (25.1)	56 (25.2)	0.154	1.688	0.822	3.464
	Primary school and less than primary school	10 (4.7)	7 (3.2)	0.117	2.500	0.796	7.853
Work type	Hard physical labour	52 (24.2)	71 (32.0)				
	Light physical labour	113 (52.6)	102 (45.9)	0.069	1.513	0.968	2.365
	Nonphysical labour	50 (23.3)	49 (22.1)	0.222	1.393	0.818	2.372
Work duration	≤1 year	5 (2.3)	6 (2.7)				
	2~ years	18 (8.4)	21 (9.5)	0.967	1.029	0.268	3.942
	4~ years	36 (16.7)	70 (31.5)	0.450	0.617	0.176	2.161
	11~ years	27 (12.6)	26 (11.7)	0.741	1.246	0.339	4.588
	16~ years	28 (13.0)	27 (12.2)	0.741	1.244	0.339	4.563
	≥21 years	101 (47.0)	72 (32.4)	0.405	1.683	0.495	5.729
Workplace	Underground	52 (24.2)	71 (32.0)				
	Underground auxiliary	68 (31.6)	69 (31.1)	0.235	1.346	0.824	2.197
	Ground	55 (25.6)	46 (20.7)	0.070	1.633	0.961	2.775
	Office workers	40 (18.6)	36 (16.2)	0.155	1.517	0.854	2.696
BMI	<18.5	1 (0.5)	11 (5.0)				
	18.5~24	55 (25.6)	94 (42.3)	0.078	6.436	0.809	51.209
	24~28	95 (44.2)	91 (41.0)	0.021^∗^	11.484	1.453	90.751
	≥28	64 (29.8)	26 (11.7)	0.002^∗^	27.077	3.325	220.507
Alcohol consumption	No	111 (51.6)	158 (71.2)				
	Yes	104 (48.4)	64 (28.8)	<0.001^∗^	2.313	1.559	3.433
Current smoking	No	89 (41.4)	124 (55.9)				
	Yes	126 (58.6)	98 (44.1)	0.003^∗^	1.791	1.226	2.617
Tea consumption	No	138 (64.2)	138 (62.2)				
	Yes	77 (35.8)	84 (37.8)	0.661	0.917	0.621	1.352
RTL	<0.57927	66 (30.7)	44 (19.8)				
	0.57927~1.45126	91 (42.3)	127 (57.2)	0.002^∗^	0.478	0.299	0.762
	>1.45126	58 (27.0)	51 (23.0)	0.311	0.758	0.444	1.296

^∗^ *P* < 0.05 and the difference is statistically significant. Abbreviations: RTL: relative telomere length; BMI: body mass index.

**Table 5:** Multifactor logistic regression of EH and other variables.

Characteristics		Case	Control	*P* value	OR	95% CI	
		*n* (%)	*n* (%)			Upper limit	Lower limit
Age	≤30	18 (8.4)	16 (7.2)				
	30~40	56 (26.0)	57 (25.7)	0.246	0.557	0.208	1.496
	40~50	79 (36.7)	76 (34.2)	0.059	0.371	0.133	1.037
	>50	62 (28.8)	73 (32.9)	0.010^∗^	0.241	0.081	0.716
Gender	Male	194 (90.2)	200 (90.1)				
	Female	21 (9.8)	22 (9.9)	0.890	0.943	0.409	2.172
Education	College or higher	16 (7.4)	28 (12.6)				
	Senior middle school	135 (62.8)	131 (59.0)	0.175	1.811	0.768	4.266
	Junior middle school	54 (25.1)	56 (25.2)	0.090	2.422	0.872	6.728
	Primary school and less than primary school	10 (4.7)	7 (3.2)	0.101	3.470	0.785	15.344
Work type	Hard physical labour	52 (24.2)	71 (32.0)				
	Light physical labour	113 (52.6)	102 (45.9)	0.390	1.336	0.691	2.582
	Nonphysical labour	50 (23.3)	49 (22.1)	0.352	1.524	0.627	3.705
Work duration	≤1 year	5 (2.3)	6 (2.7)				
	2~ years	18 (8.4)	21 (9.5)	0.842	0.861	0.196	3.779
	4~ years	36 (16.7)	70 (31.5)	0.687	0.749	0.183	3.060
	11~ years	27 (12.6)	26 (11.7)	0.634	1.429	0.328	6.227
	16~ years	28 (13.0)	27 (12.2)	0.697	1.343	0.305	5.917
	≥21 years	101 (47.0)	72 (32.4)	0.181	2.605	0.641	10.588
Workplace	Underground	52 (24.2)	71 (32.0)				
	Underground auxiliary	68 (31.6)	69 (31.1)	0.429	1.310	0.672	2.554
	Ground	55 (25.6)	46 (20.7)	0.332	1.466	0.677	3.175
	Office workers	40 (18.6)	36 (16.2)	0.220	1.781	0.709	4.475
BMI	<18.5	1 (0.5)	11 (5.0)				
	18.5~24	55 (25.6)	94 (42.3)	0.086	6.659	0.766	57.906
	24~28	95 (44.2)	91 (41.0)	0.032^∗^	10.580	1.222	91.600
	≥28	64 (29.8)	26 (11.7)	0.004^∗^	23.914	2.682	213.214
Alcohol consumption	No	111 (51.6)	158 (71.2)				
	Yes	104 (48.4)	64 (28.8)	0.002^∗^	2.103	1.311	3.375
Current smoking	No	89 (41.4)	124 (55.9)				
	Yes	126 (58.6)	98 (44.1)	0.014^∗^	1.812	1.126	2.916
Tea consumption	No	138 (64.2)	138 (62.2)				
	Yes	77 (35.8)	84 (37.8)	0.037^∗^	0.609	0.383	0.970
RTL	<0.57927	66 (30.7)	44 (19.8)				
	0.57927~1.45126	91 (42.3)	127 (57.2)	0.018^∗^	0.529	0.312	0.897
	>1.45126	58 (27.0)	51 (23.0)	0.551	0.827	0.443	1.544

^∗^ *P* < 0.05 and the difference is statistically significant. Abbreviations: RTL: relative telomere length; BMI: body mass index.

## Data Availability

Data used to support the findings of this study are available from the corresponding author upon reasonable request.
